# Disease burden and rehabilitation needs of preterm newborns in China from 1990 to 2021: trend analysis, factor decomposition, attributable risk factors, and projections toward 2035

**DOI:** 10.3389/fpubh.2025.1674472

**Published:** 2025-12-03

**Authors:** Mengchen Zhu, Shuhua Fan, Shujuan Zhang, Limei Yang

**Affiliations:** 1 The First Clinical Medical College, Lanzhou University, Lanzhou, Gansu, China; 2 Women and Children’s Hospital, Central Hospital of Zhumadian, Family-Centered Birthing Center, Zhumadian, China

**Keywords:** preterm birth, rehabilitation needs, disease burden, risk factors, decomposition analysis, global burden of disease study

## Abstract

**Objective:**

To analyze trends, risk factors, and project future trajectories of rehabilitation needs and disease burden in preterm newborns in China (1990–2035).

**Methods:**

Using GBD 2021 data, we assessed preterm birth burden via age-standardized prevalence rate (ASPR) and age-standardized years lived with disability rate (ASYR). Joinpoint regression calculated annual percentage changes (APC, AAPC) and estimated annual percentage changes (EAPC). Decomposition analysis quantified contributions of aging, population growth, and epidemiological changes to YLDs. Risk factor attribution was assessed for low birth weight, short gestation, ambient particulate matter pollution, and household air pollution from solid fuels. An ARIMA model projected trends to 2035.

**Results:**

ASPR declined significantly from 812.7/100,000 (95% CI: 702.2–931.2) in 1990 to 645.4/100,000 (95% CI: 572.5–728.1) in 2021 (EAPC = −1.1, 95% CI: −1.3 to −0.9). ASYR decreased from 67.4/100,000 (95% CI: 48.1–89.1) to 63.1/100,000 (95% CI 45.2–80.7) (EAPC = −0.5, 95% CI: −0.6 to −0.5). Males exhibited higher ASPR and ASYR than females. Low birth weight and short gestation were predominant risk factors (e.g., ASYR attributable to low birth weight: 67.43/100,000 in 1990, 63.06/100,000 in 2021). Decomposition showed epidemiological changes (−707,840.1% for low birth weight/short gestation) and population growth (718,149.34%) drove YLD changes. Projections estimate preterm births decreasing from 7,887,971 (2022) to 7,568,882 (2035), with ASPR falling to 506.88/100,000 and ASYR potentially rising slightly to 60.45/100,000 by 2035.

**Conclusion:**

While preterm birth burden in China has decreased since 1990, a significant burden persists, disproportionately affecting males. Low birth weight and short gestation are key modifiable risks. Projected trends underscore the need for enhanced public health interventions targeting identified risk factors and strengthening continuum-of-care rehabilitation services to further reduce burden and improve outcomes.

## Introduction

Preterm birth is still an important global health problem, which is the main reason for the significant increase of neonatal incidence rate, mortality and long-term complications. Preterm babies have a higher risk of complications than full-term babies, leading to a range of short-term health problems such as respiratory distress syndrome, hypoglycemia, hypothermia, jaundice, feeding difficulties and infections (1), as well as long-term health problems such as cerebral palsy, epilepsy, and blindness. Approximately 13.4 million newborns are born prematurely worldwide each year, accounting for about one tenth of all live births. Shockingly, nearly one million preterm births die each year (2, 3). Research shows that preterm birth mortality accounts for 40% of newborn deaths, which is considered the leading cause of death for children under five years old worldwide and the most common cause of perinatal death (4). With a large population and a high birth rate, China is the second largest preterm birth country in the world (5). With the rapid development of perinatal medicine and neonatal intensive care technology, more and low birth weight infants are able to survive, but with it comes an urgent need for high-quality, continuous, personalized nursing and rehabilitation support.

Nursing management, as a possible non pharmacological approach, improves perinatal outcomes by addressing many potential socio-economic factors that plague preterm birth and other adverse pregnancy outcomes (6). These challenges not only exist in the neonatal intensive care unit (NICU) during hospitalization, but also extend to the home and community environment after discharge (7). Therefore, a comprehensive and in-depth understanding and satisfaction of the nursing and rehabilitation needs of preterm birth children is a key link in reducing their mortality and disability rates, optimizing their long-term quality of life, and is also a core issue currently faced by neonatal medicine, rehabilitation medicine, child health care, and family support services. Further research is urgently needed to develop appropriate and effective prevention strategies and intervention measures to improve the survival rate of preterm born newborns; and expand known effective interventions to improve the health of preterm born children.

Despite various measures being implemented to reduce the incidence and mortality of premature birth, it remains a significant social challenge. The 2021 Global Burden of Disease highlights new and existing health threats that need to be prioritized on the international public health agenda. Previous research has reported on the enormous burden of premature birth worldwide, emphasizing the urgency of timely attention and dedicated efforts to address this global challenge. Although GBD research has made progress in quantifying the disease burden of premature infants in the acute phase, there is still a significant research gap in systematically evaluating the long-term rehabilitation needs of premature infants, lacking exploration of risk factors and future trends for premature birth in China, and not combining decomposition analysis to understand the driving factors behind changes in these indicators. By utilizing the systematic data from the GBD database, this gap can be filled, and a comprehensive understanding of the latest epidemiological characteristics and burden related risk factors of premature birth in China can be revealed, which can promote more scientific and rational policy-making. Predicting the future trend of premature birth disease burden to develop specific policies and plans, in order to better understand and alleviate the burden of premature birth in China. Previous studies have focused more on the incidence, mortality, and prevalence of risk factors for preterm birth, while there is relatively insufficient comprehensive evaluation of the nursing and rehabilitation needs, disease burden, and attributable risk factors of preterm birth patients in China. Therefore, based on data from the 2021 Global Burden of Disease Study (GBD) (8), this study establishes a three-dimensional analytical framework encompassing “disease burden–care and rehabilitation needs–risk factors” to systematically assess the trends, attributable risk factors, and projections toward 2035 for the rehabilitation needs and disease burden of preterm birth in China. The findings will provide a scientific basis for formulating preterm birth prevention and control strategies, optimizing healthcare resource allocation, and improving patients’ quality of life in China.

## Methods

### Data sources

Data from GBD 2021 results^1^. This is the largest and latest comparative assessment of the burden of 371 diseases and injuries at the global, regional, and national levels. According to the World Health Organization’s (WHO) GBD study, preterm birth is defined as a baby born alive before 37 weeks of pregnancy. Bayesian Meta Regression Disease Modeling Meta Regression (DisMod MR, version 2.1) is a Bayesian meta regression framework applied to GBD data modeling. GBD 2021 data sources include population censuses, disease registrations, hospital records, epidemiological surveys, and published literature (9, 10). This study extracted epidemiological data of preterm births in China stratified by age and gender from 1990 to 2021, including the number of cases, years lived with disability (YLDs), and attributable risk factors (including short pregnancy, environmental particulate matter pollution, household air pollution caused by solid fuels, and low birth weight) (11).

### Definition and calculation of nursing rehabilitation needs and disease burden indicators

Disease incidence: defined as the number of cases in preterm birth patients who require rehabilitation interventions (including diagnosis and treatment).

Estimation of Prevalence: Using GBD 2021 data, calculate the prevalence of preterm birth in China. The incidence rate is defined as the number of preterm birth patients per 100,000 population (8, 11–13).

Age standardized prevalence rate (ASPR): Refers to the prevalence rate adjusted according to the age structure of a specific standard population. This study used the World Health Organization (WHO) standard population (2000–2025) for age standardization to eliminate the impact of age structure differences in different years or populations on the incidence rate, thereby more accurately comparing preterm birth prevalence levels between different time periods or populations. The unit is per 100,000 population (8, 11–13).

YLDs: Refers to the years of healthy life lost due to preterm birth, reflecting the impact of preterm birth on patients’ quality of life. YLDs = number of illnesses multiplied by disability weight (DW), where DW is determined by GBD research based on expert evaluations and population surveys to quantify the severity of different health states, ranging from 0 (perfect health) to 1 (death), representing the severity of the disease (8, 11–13).

Age standardized YLD rate (ASYR): refers to the YLD rate adjusted according to the age structure of a specific standard population. Similar to ASPR, ASYR is used to eliminate the impact of age structure differences on YLDs, in order to more accurately compare preterm birth disease burden between different time periods or populations. The unit is per 100,000 population (8, 11–13).

### Attributable risk factors

The main estimated attributable burden of three risk factors associated with preterm birth is: low birth weight, short pregnancy period, environmental particulate matter pollution, and household air pollution caused by solid fuels. Data on the impact of these risk factors were obtained from the GBD 2021, which quantifies risk factor contributions using Comparative Risk Assessment (CRA) methods. This involves calculating population-attributable fractions (PAFs) by integrating exposure prevalence, relative risks from epidemiological studies, and theoretical minimum risk exposure levels (TMRELs) to estimate the proportion of burden attributable to each risk factor.

### Decomposition analysis

Decomposition analysis can provide a deeper understanding of the specific reasons for the changes in YLDs caused by risk factors within a specified time period (14). This study further explored the impact of potential factors on the epidemiology of YLDs through decomposition analysis. Decompose the changes in YLDs caused by preterm birth into aging, population, and epidemiological changes, in order to quantify the impact of these factors on overall YLDs (15, 16).

### Preterm newborns predict

This study uses the ARIMA model to predict the future burden of premature birth in China from 2022 to 2035. The ARIMA model is widely used for predicting time series data due to its ability to capture trends, seasonality, and potential patterns in disease burden. In previous studies, ARIMA has been effectively used to predict the incidence rate and economic burden of chronic diseases (such as chronic kidney disease, hand foot mouth disease and other infectious diseases). Given its powerful predictive ability, this study used ARIMA to predict the future burden of premature birth in China. This model can explain the past trends and fluctuations of diseases, making it a suitable choice for generating reliable predictions, which is crucial for health policy planning, resource allocation, and preventive interventions (17).

### Statistical analysis

Analyze the time trend of preterm birth burden through Joinpoint regression (Version 4.9.0), and then fit the simplest model to the data by connecting several different line segments on a logarithmic scale (18). The Joinpoint model uses a structured approach to evaluate temporal trends and determine the most suitable connections for statistically significant trend changes (19, 20). Annual percentage change (APC) and its 95% confidence interval (CI) are calculated using the geometric weighted average of various annual percentage change values in regression analysis, and are used to estimate the time trend of prevalence and YLDs (21). Average annual percentage change (AAPC) is a weighted average of trend summaries and APCs over a predetermined fixed time interval, used to describe the average APC over multiple years. Calculate the estimated annual percentage change (EAPC) to evaluate the trend of ASPR and ASYR changes over time in preterm newborns. EAPC originates from linear regression models, where the natural logarithm of ASPR or ASYR is modeled based on time (years). When EAPC is greater than zero and *p* < 0.05, it is determined to show an upward trend. On the contrary, when EAPC is less than zero and *p* < 0.05, it indicates a downward trend. The calculation formula for EAPC is as follows: EAPC = 100 × (exp (*β*) −1), where *β* is the regression coefficient for linear regression of the number of diseases, YLDs, ASPR, and ASYR with respect to the year (22–25). Gender and age stratification analysis: Conduct gender and age stratification analysis on the incidence, YLDs, ASPR, and ASYR of preterm birth in newborns to evaluate the differences between different gender and age groups. The use of decomposition analysis intuitively demonstrated the role of three factors (i.e., aging, population, and epidemiology) that drove changes in YLDs between 1990 and 2021. Using ARIMA model to simulate and generate predicted values for the years 2022–2035, all analyses were completed using R language (version 4.3.1).

## Results

### Overall trends for preterm birth disease burden and rehabilitation needs in Chinese newborns

**Figure 1:**
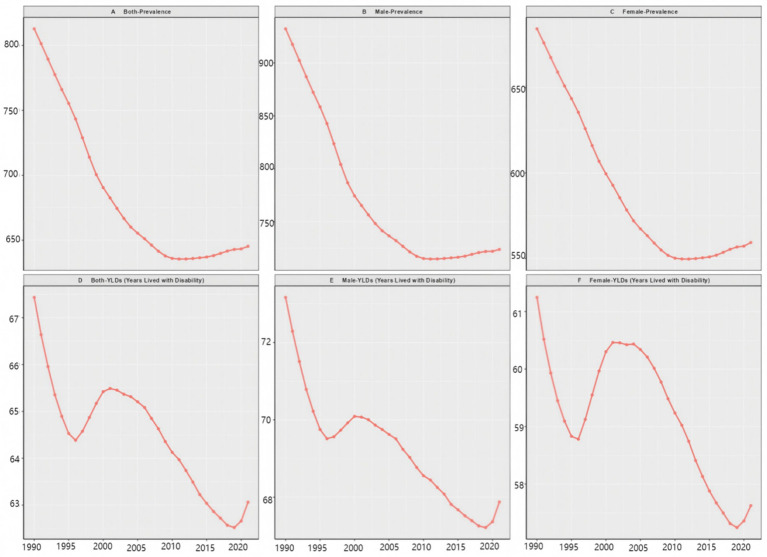
Trend chart of ASPR and ASYR for preterm births in Chinese newborns from 1990 to 2021.

In 1990, there were 9,498,917 (95% UI: 8,196,798–10,897,739) preterm birth patients in China who required rehabilitation services, resulting in 824,193 (95% UI: 587,355–1,089,548) YLDs (Figure 1; Table 1). By 2021, the number of cases had decreased to 7,907,240 (95% UI: 6,878,507–9,093,866), and YLDs had increased to 832,784 (95% UI: 595,325–1,066,130). ASPR decreased from 812.7/100,000 (95% CI: 702.2–931.2) in 1990 to 645.4/100,000 (95% CI: 572.5–728.1) in 2021, while ASYR decreased from 67.4/100,000 (95% CI: 48.1–89.1) to 63.1/100,000 (95% CI: 45.2–80.7). Between 1990 and 2021, the ASPR and ASYR of preterm birth showed a downward trend, while the EAPC of ASPR was −1.1% (95% CI: −1.3–0.9) and the EAPC of ASYR was −0.5% (95% CI: −0.6–0.5) (see Table 1). Male ASPR decreased from 932.2/100,000 (95% CI: 803.7–1,068.1) in 1990 to 723.9/100,000 (95% CI: 641.6–818.7) in 2021, while ASYR decreased from 73.2/100,000 (95% CI: 52.4–96.7) to 67.9/100,000 (95% CI: 48.7–86.8). The EAPC of ASPR is −1.1% (95% CI: −1.3–0.9), while the EAPC of ASYR is −0.6% (95% CI: −0.5–0.7). Female ASPR decreased from 684.5/100,000 (95% CI: 595.1–786.9) in 1990 to 559.3723.9/100,000 (95% CI: 497.5–630) in 2021, while ASYR decreased from 61.2/100,000 (95% CI: 43.5–80.5) to 57.6/100,000 (95% CI: 40.9–74.2). The EAPC of ASPR is −1.0% (95% CI: −1.1–0.8), while the EAPC of ASYR is −0.4% (95% CI: −0.5–0.3).

**Table 1:** Prevalence, standardized rates, and estimated annual percentage changes (EAPC) of preterm births in Chinese newborns from 1990 to 2021.

Gender	Prevalence in 1990 (95% UI)	ASPR in 1990 (95% UI)	Prevalence in 2021 (95% UI)	ASPR in 2021 (95% UI)	EAPC (95% CI)	YLDs in 1990 (95% UI)	ASYP in 1990 (95% UI)	YLDs in 2021 (95% UI)	ASYP in 2021 (95% UI)	EAPC (95% CI)
Total	9,498,917 (8,196,798–10,897,739)	812.7 (702.2–931.2)	7,907,240 (6,878,507–9,093,866)	645.4 (572.5–728.1)	−1.1 (−1.3–−0.9)	824,193 (587,355–1,089,548)	67.4 (48.1–89.1)	832,784 (595,325–1,066,130)	63.1 (45.2–80.7)	−0.5 (−0.6–−0.5)
Male	5,662,675 (4,875,964–6,491,905)	932.2 (803.7–1,068.1)	4,573,579 (3,973,005–5,271,404)	723.9 (641.6–818.7)	−1.1 (−1.3–−0.9)	464,455 (332,740–615,396)	73.2 (52.4–96.7)	457,648 (327,457–585,184)	67.9 (48.7–86.8)	−0.6 (−0.5–−0.7)
Female	3,836,242 (3,323,712–4,420,913)	684.5 (595.1–786.9)	3,333,661 (2,909,020–3,821,376)	559.3 (497.5–630)	−1.0 (−1.1–−0.8)	359,738 (255,950–473,394)	61.2 (43.5–80.5)	375,136 (267,029–484,835)	57.6 (40.9–74.2)	−0.4 (−0.5–−0.3)

### Joinpoint regression analysis of preterm birth disease burden and rehabilitation needs in Chinese newborns

**Figure 2:**
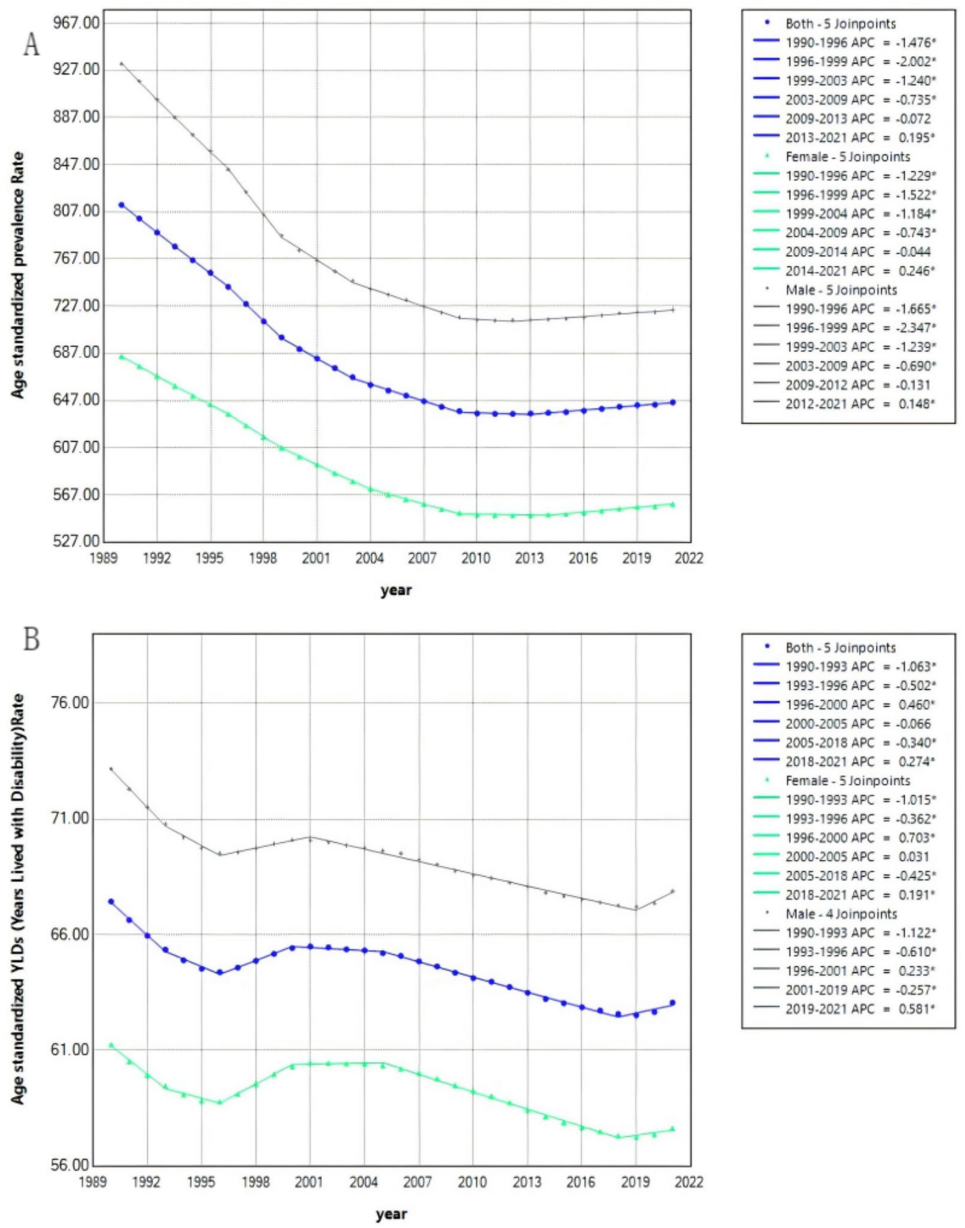
Joinpoint regression analysis of preterm birth nursing rehabilitation needs and disease-specific age standardized rates for newborns in China from 1990 to 2021. **(A)** Age standardized prevalence rate. **(B)** Age standardized YLDs rate.

The Joinpoint regression analysis results of ASPR and ASYR for the rehabilitation needs and disease burden of Chinese newborns from 1990 to 2021 are shown in Figure 2 and Table 2. During the period of 1990–2021, the ASPR (AAPC = −0.74%) of newborns with preterm birth showed a downward trend, with the largest decrease observed from 1996 to 1999 (APC = −2.00%). The decrease in ASPR (AAPC = −0.82%) of males was higher than that of females (AAPC = −0.65%), with females experiencing the largest decrease from 1996 to 1999 (APC = −1.52%) and males experiencing the largest increase from 1996 to 1999 (APC = −2.35%); the ASYR (AAPC = −0.22%) of newborns with preterm birth showed a downward trend, with the largest decrease from 1990 to 1993 (APC = −1.06%). The decrease in ASYR (AAPC = −0.24%) for males was higher than that for females (AAPC = −0.20%), with females experiencing the largest decrease from 1990 to 1996 (APC = −1.01%) and males experiencing the largest decrease from 1990 to 1996 (APC = −1.12%).

**Table 2:** Joinpoint regression analysis of preterm birth nursing rehabilitation needs and disease burden in Chinese newborns from 1992 to 2021.

ASPR					ASYR				
Time period	APC	95% CI	*t*-values	*p*	Time period	APC	95% CI	*t*-values	*p*
1990–1996	−1.48	−1.51–−1.45	−103.55	< 0.001	1990–1993	−1.06	−1.17–−0.95	−20.58	< 0.001
1996–1999	−2.00	−2.16–−1.84	−26.78	< 0.001	1993–1996	−0.50	−0.72–−0.28	−4.91	< 0.001
1999–2003	−1.24	−1.32–−1.16	−34.56	< 0.001	1996–2000	0.46	0.35–0.57	9.22	< 0.001
2003–2009	−0.74	−0.77–−0.70	−46.36	< 0.001	2000–2005	−0.07	−0.13-0	−2.1	0.054
2009–2013	−0.07	−0.15-0.00	−2.00	0.064	2005–2018	−0.34	−0.35–−0.33	−56.44	< 0.001
2013–2021	0.19	0.18–0.21	24.79	< 0.001	2018–2021	0.27	0.17–0.38	5.55	< 0.001
1990–2021	−0.74*	−0.76–−0.72	−68.73	< 0.001	1990–2021	−0.22*	−0.25–−0.19	−14.84	< 0.001
1990–1996	−1.23	−1.26–−1.20	−84.49	< 0.001	1990–1993	−1.01	−1.13–−0.9	−18.65	< 0.001
1996–1999	−1.52	−1.68–−1.36	−20.18	< 0.001	1993–1996	−0.36	−0.59–−0.13	−3.36	0.004
1999–2004	−1.18	−1.23–−1.14	−51.78	< 0.001	1996–2000	0.70	0.59–0.82	13.27	< 0.001
2004–2009	−0.74	−0.79–−0.69	−32.55	< 0.001	2000–2005	0.03	−0.04-0.1	0.93	0.366
2009–2014	−0.04	−0.09-0.00	−1.97	0.068	2005–2018	−0.43	−0.44–−0.41	−66.84	< 0.001
2014–2021	0.25	0.23–0.27	25.67	< 0.001	2018–2021	0.19	0.08–0.3	3.65	0.002
1990–2021	−0.65*	−0.67–−0.63	−62.90	< 0.001	1990–2021	−0.20*	−0.23–−0.17	−12.69	< 0.001
1990–1996	−1.67	−1.70–−1.63	−107.33	< 0.001	1990–1993	−1.12	−1.26–−0.99	−17.16	< 0.001
1996–1999	−2.35	−2.52–−2.17	−28.35	< 0.001	1993–1996	−0.61	−0.88–−0.34	−4.77	< 0.001
1999–2003	−1.24	−1.32–−1.16	−31.20	< 0.001	1996–2001	0.23	0.15–0.32	5.83	< 0.001
2003–2009	−0.69	−0.73–−0.65	−39.32	< 0.001	2001–2019	−0.26	−0.27–−0.25	−57.57	< 0.001
2009–2012	−0.13	−0.30-0.04	−1.65	0.120	2019–2021	0.58	0.32–0.85	4.62	< 0.001
2012–2021	0.15	0.13–0.16	20.44	< 0.001					
1990–2021	−0.82*	−0.84–−0.79	−61.67	< 0.001	1990–2021	−0.24*	−0.28–−0.21	−13.82	< 0.001

* refers to AAPC.

### Attribution analysis of risk factors

**Figure 3:**
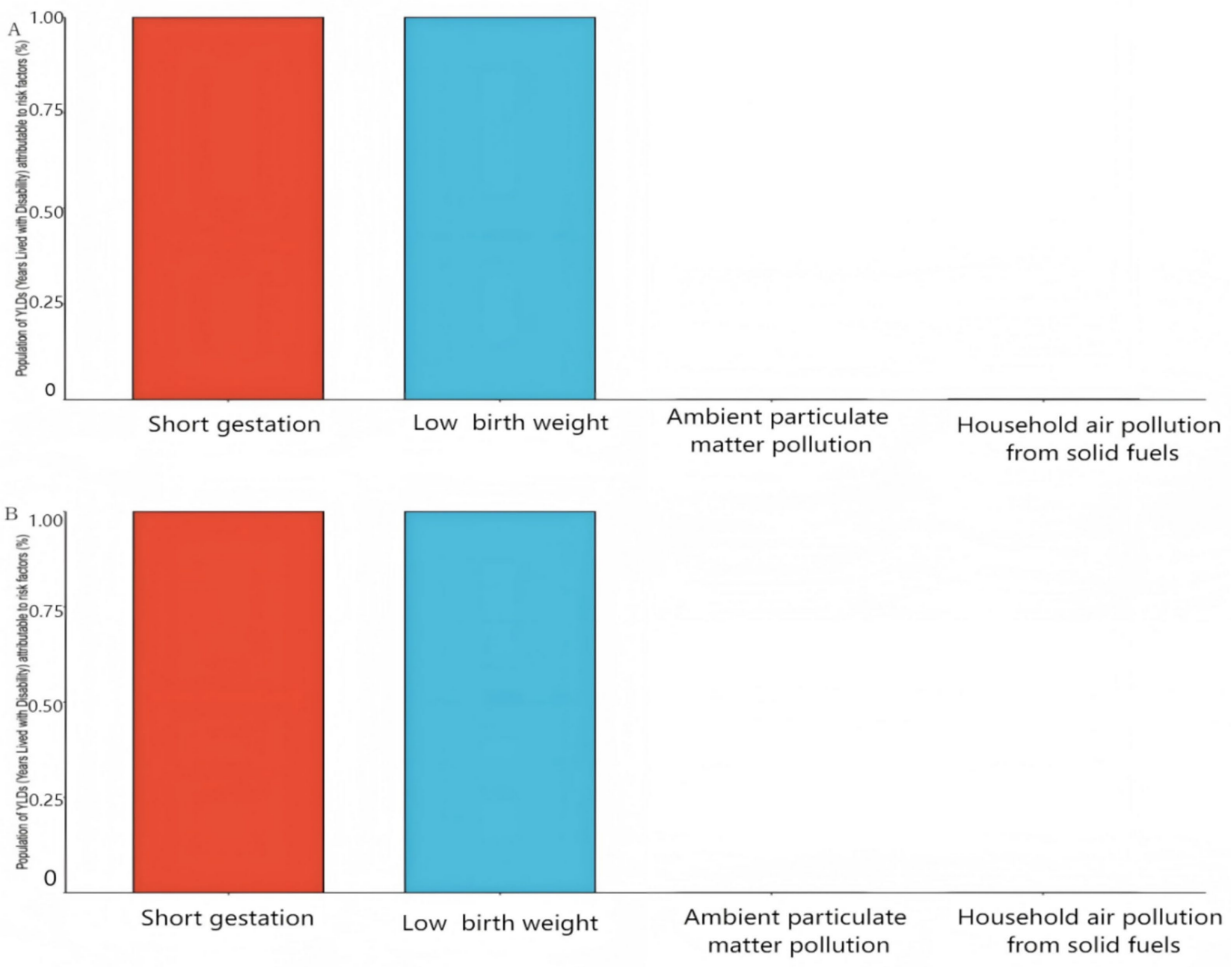
Risk factors for preterm birth YLDs in Chinese newborns from 1990 to 2021.

From 1990 to 2021, low birth weight, short pregnancy period, environmental particulate matter pollution, and household air pollution caused by solid fuels were the main risk factors for the burden of preterm birth disease in newborns (Figure 3). The disease burden caused by low birth weight and short pregnancy period remains relatively stable, with only a 1.04% increase, and remains an important risk factor. The environmental risk factors have undergone significant changes: the disease burden caused by household air pollution has decreased significantly (−78.96%), ranking second from “environmental particulate matter”; At the same time, the disease burden of environmental particulate matter (such as air pollution) has decreased significantly (−84.78%), rising from third place to first place.

The number of YLDs related to environmental particulate matter pollution decreased from 264 cases (95% CI: 120–521) in 1990 to 208 cases (95% CI: 106–302) in 2021 (Table 3), and ASYR showed a slow upward trend (EAPC = 2.24, 95% CI: 1.98–2.49). The growth rate of women (EAPC = 2.36%) is significantly higher than that of men (EAPC = 2.15%) (Table 3). The YLDs related to household air pollution caused by solid fuels decreased the most, from 1,371 cases (95% CI: 925–1,885) to 55 cases (95% CI: 12–151), with an average annual decrease of 8.16% (95% CI: −8.75–7.56) in ASYR. The decrease in males (EAPC = −8.29%) was significantly higher than that in females (EAPC = −7.97%). The increase in YLDs related to low birth weight was the largest, increasing from 824,193 cases (95% CI: 587,355–1,089,548) to 832,784 cases (95% CI: 595,325–1,066,130). The average annual decrease in ASYR was −0.18% (95% CI: −0.21–0.14), and the decrease in males (EAPC = −0.21%) was significantly higher than that in females (EAPC = −0.15%). The increase in YLDs related to short pregnancy period was the largest, from 824,193 cases (95% CI: 587,355–1,089,548) to 832,784 cases (95% CI: 595,325–1,066,130). The average annual decrease in ASYR was −0.18% (95% CI: −0.21–0.14), and the decrease in male (EAPC = −0.21%) was significantly higher than that in female (EAPC = −0.15%).

**Table 3:** Trend of preterm birth YLDs in newborns caused by risk factors in China from 1990 to 2021.

Gender	YLDs in 1990 (95% UI)	ASYP in 1990 (95% UI)	YLDs in 2021 (95% UI)	ASYP in 2021 (95% UI)	EAPC (95% CI)
Both					
Ambient particulate matter pollution	264 (120–521)	0.02 (0.01–0.05)	208 (106–302)	0.04 (0.02–0.06)	2.24 (1.98–2.49)
Household air pollution from solid fuels	1,371 (925–1,885)	0.12 (0.08–0.17)	55 (12–151)	0.01 (0–0.03)	−8.16 (−8.75–−7.56)
Low birth weight	824,193 (587,355–1,089,548)	67.43 (48.07–89.11)	832,784 (595,325–1,066,130)	63.06 (45.22–80.65)	−0.18 (−0.21–−0.14)
Short gestation	824,193 (587,355–1,089,548)	67.43 (48.07–89.11)	832,784 (595,325–1,066,130)	63.06 (45.22–80.65)	−0.18 (−0.21–−0.14)
Male					
Ambient particulate matter pollution	158 (72–309)	0.03 (0.01–0.05)	120 (61–174)	0.04 (0.02–0.06)	2.15 (1.88–2.42)
Household air pollution from solid fuels	820 (555–1,130)	0.14 (0.09–0.19)	31.81 (6–86)	0.01 (0–0.03)	−8.29 (−8.89–−7.68)
Low birth weight	464,455 (332,740–615,396)	73.17 (52.39–96.72)	457,648 (327,457–585,184)	67.88 (48.73–86.82)	−0.21 (−0.24–−0.18)
Short gestation	464,455 (332,740–615,396)	73.17 (52.39–96.72)	457,648 (327,457–585,184)	67.88 (48.73–86.82)	−0.21 (−0.24–−0.18)
Female					
Ambient particulate matter pollution	105 (48–207)	0.02 (0.01–0.04)	87 (45–127)	0.04 (0.02–0.05)	2.36 (2.12–2.61)
Household air pollution from solid fuels	550 (367–761)	0.11 (0.07–0.15)	23 (5–63)	0.01 (0–0.03)	−7.97 (−8.56–−7.38)
Low birth weight	359,738 (255,950–473,394)	61.25 (43.49–80.54)	375,136 (267,028–484,835)	57.63 (40.94–74.21)	−0.15 (−0.2–−0.1)
Short gestation	359,738 (255,950–473,394)	61.25 (43.49–80.54)	375,136 (267,028–484,835)	57.63 (40.94–74.21)	−0.15 (−0.2–−0.1)

### Decomposition analysis

**Figure 4:**
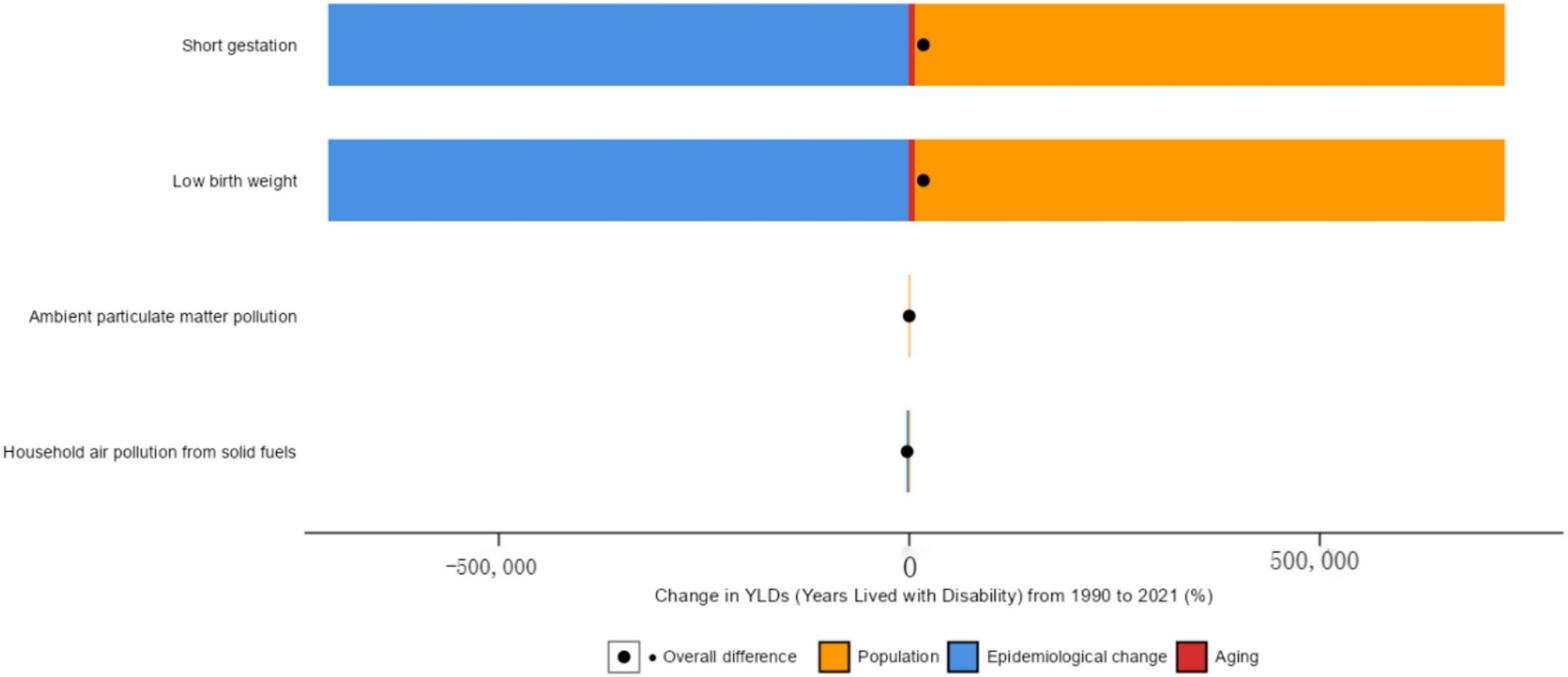
Relative contributions of aging, population, and epidemiological changes to ASYR from 1990 to 2021.

Using decomposition analysis to quantify the relative contributions of changes in age structure, population, and epidemiology to the overall burden of preterm birth in Chinese newborns (Figure 4; Table 4). The results show the decomposition analysis of YLDs changes for three demographic determinants (aging, population, and epidemiological changes). The preterm birth decomposition analysis of newborns caused by short pregnancy period shows that 718,149.34% of YLDs changes are related to population, followed by epidemiological changes (−707,840.1%) and aging (6,872.83%). The preterm birth decomposition analysis of newborns caused by environmental particulate matter pollution shows that −296.83% of YLDs changes are related to epidemiological changes, followed by population (230.20%) and aging (−44.42%). The preterm birth decomposition analysis of newborns caused by household air pollution caused by solid fuels shows that −2,773.19% of YLDs changes are related to epidemiological changes, followed by population (1,194.64%) and aging (−1,052.37%). The preterm birth decomposition analysis of newborns caused by low birth weight shows that 718149.34% of YLDs changes are related to population, followed by epidemiological changes (−707,840.1%) and aging (6,872.83%). The contribution of epidemiological changes to YLDs is negative in all preterm births caused by risk factors, while the contribution of population to YLDs is positive.

**Table 4:** Relative contributions of aging, population, and epidemiological changes to YLDs from 1990 to 2021.

Risk factors	Population (%)	Aging (%)	Epidemiological changes (%)	Net change (%)
Low birth weight	718,149.34	6,872.83	−707,840.1	17,182.07
Ambient particulate matter pollution	230.20	−44.42	−296.83	−111.06
Household air pollution from solid fuels	1,194.64	−1,052.37	−2,773.19	−2,630.92
Short gestation	718,149.34	6,872.83	−707,840.1	17,182.07

### Prediction of preterm birth disease burden trends in Chinese newborns

**Figure 5:**
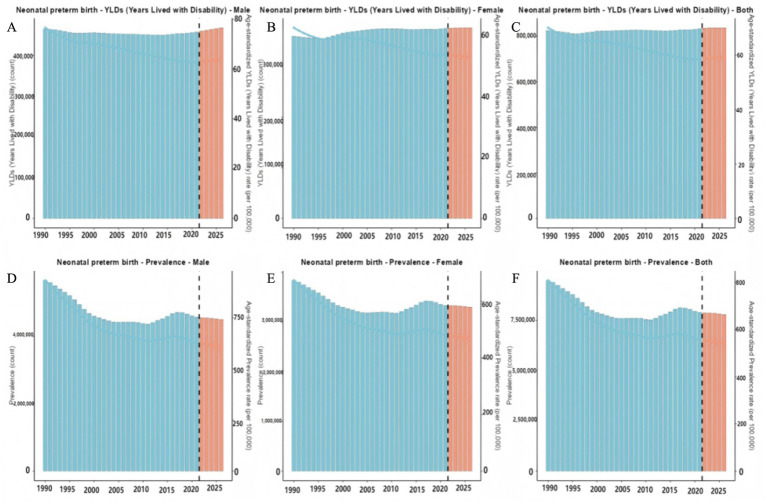
ARIMA model predictions for the rehabilitation needs in preterm birth. **(A)** The years lived with disability (YLDs) cases and rate among male predicted by the ARIMA model; **(B)** The YLDs cases and rate among female predicted by the ARIMA model; **(C)** The YLDs cases and rate among overall population predicted by the ARIMA model; **(D)** The prevalence cases and rate among male predicted by the ARIMA model; **(E)** The prevalence cases and rate among female predicted by the ARIMA model; **(F)** The prevalence cases and rate among overall population predicted by the ARIMA model.

The demand for preterm birth rehabilitation needs in Chinese newborns, as well as the number of diseases, ASPR, YLDs, and ASYR predicted in the future, are shown in Figure 5 and Table 5. The number of preterm birth diseases in Chinese newborns is expected to decrease from 7,887,971 in 2022 to 7,568,882 in 2035. The number of male diseases is expected to decrease from 4,558,913 in 2022 to 4,346,704 in 2035, and the number of female diseases is expected to decrease from 3,329,040 in 2022 to 3,221,876 in 2035. The number of male diseases is higher than that of female diseases; ASPR is expected to decrease from 553.24/100,000 in 2022 to 506.88/100,000 in 2035, male ASPR is expected to decrease from 624.73/100,000 in 2022 to 566.52/100,000 in 2035, and female ASPR is expected to decrease from 478.28/100,000 in 2022 to 443.95/100,000 in 2035. The number of preterm births YLDs in newborns in China is expected to increase from 834,978 in 2022 to 835,966 in 2035. The number of YLDs in males is expected to increase from 459,681 in 2022 to 486,119 in 2035, and the number of YLDs in females is expected to increase from 375,730 in 2022 to 376,568 in 2035. The number of YLDs in males is higher than that in females; ASYR is expected to increase from 58.65/100,000 in 2022 to 60.45/100,000 in 2035, with male ASYR expected to increase from 63.04/100,000 in 2022 to 66.15/100,000 in 2035, and female ASYR expected to decrease from 53.99/100,000 in 2022 to 50.24/100,000 in 2035.

**Table 5:** Preterm birth nursing rehabilitation needs and disease incidence, ASPR, YLDs, and ASYR predictions for newborns in China from 2022 to 2035.

Year	Prevalence (95% CI)	ASPR/100,000 (95% CI)	YLDs (95% CI)	ASYR/100,000 (95% CI)
Male				
2022	4,558,913 (4,533,977–4,583,849)	624.73 (621.35–628.12)	459,681 (458,848–460,514)	63.04 (62.90–63.19)
2023	4,552,414 (4,479,697–4,625,131)	621.96 (612.17–631.75)	461,715 (459,853–463,577)	63.27 (62.89–63.64)
2024	4,541,880 (4,406,427–4,677,333)	618.10 (600.09–636.10)	463,749 (460,632–466,865)	63.50 (62.81–64.19)
2025	4,524,749 (4,319,642–4,729,856)	613.16 (586.24–640.09)	465,782 (461,220–470,344)	63.74 (62.68–64.80)
2026	4,504,527 (4,226,297–4,782,756)	607.98 (571.83–644.13)	467,816 (461,639–473,993)	63.98 (62.49–65.48)
2027	4,484,936 (4,129,940–4,839,932)	603.11 (557.29–648.92)	469,850 (461,904–477,795)	64.22 (62.253–66.19)
2028	4,467,181 (4,030,444–4,903,918)	598.59 (542.43–654.74)	471,883 (462,028–481,739)	64.46 (61.96–66.96)
2029	4,450,503 (3,926,229–4,974,777)	594.16 (526.87–661.45)	473,917 (462,020–485,814)	64.70 (61.64–67.77)
2030	4,433,809 (3,816,272–5,051,346)	589.64 (510.48–668.80)	475,951 (461,889–490,012)	64.94 (61.28–68.61)
2031	4,416,627 (3,700,646–5,132,609)	585.01 (493.34–676.69)	477,984 (461,641–494,328)	65.19 (60.88–69.49)
2032	4,399,092 (3,580,014–5,218,170)	580.35 (475.59–685.11)	480,018 (461,282–498,754)	65.43 (60.45–70.41)
2033	4,381,511 (3,454,988–5,308,033)	575.71 (457.33–694.09)	482,052 (460,816–503,287)	65.67 (59.98–71.35)
2034	4,364,052 (3,325,870–5,402,234)	571.11 (438.57–703.65)	484,085 (460,249–507,922)	65.91 (59.49–72.33)
2035	4,346,704 (3,192,732–5,500,676)	566.52 (419.30–713.74)	486,119 (459,583–512,655)	66.15 (58.97–73.34)
Female				
2022	3,329,040 (3,313,226–3,344,854)	478.28 (476.03–480.53)	375,730 (375,043–376,417)	53.99 (53.86–54.11)
2023	3,328,837 (3,283,633–3,374,040)	476.95 (470.56–483.35)	376,139 (374,447–377,831)	53.92 (53.58–54.27)
2024	3,323,891 (3,241,383–3,406,398)	474.63 (463.06–486.20)	376,393 (373,518–379,268)	53.79 (53.17–54.42)
2025	3,313,740 (3,190,927–3,436,552)	471.51 (454.45–488.57)	376,535 (372,433–380,637)	53.60 (52.65–54.54)
2026	3,302,198 (3,137,562–3,466,834)	468.34 (445.64–491.05)	376,602 (371,311–381,894)	53.34 (52.06–54.62)
2027	3,292,056 (3,083,371–3,500,741)	465.51 (436.85–494.16)	376,625 (370,224–383,026)	53.03 (51.43–54.64)
2028	3,283,490 (3,027,514–3,539,467)	462.92 (427.84–498.00)	376,624 (369,206–384,043)	52.69 (50.79–54.60)
2029	3,275,362 (2,968,498–3,582,227)	460.34 (418.32–502.35)	376,614 (368,270–384,957)	52.34 (50.16–54.52)
2030	3,266,821 (2,905,750–3,627,891)	457.64 (408.24–507.04)	376,601 (367,415–385,786)	51.97 (49.55–54.40)
2031	3,257,802 (2,839,652–3,675,952)	454.87 (397.71–512.03)	376,589 (366,632–386,546)	51.61 (48.97–54.25)
2032	3,248,646 (2,770,831–3,726,462)	452.10 (386.84–517.36)	376,580 (365,911–387,249)	51.25 (48.42–54.08)
2033	3,239,612 (2,699,646–3,779,579)	449.37 (375.67–523.07)	376,574 (365,242.–387,906)	50.91 (47.91–53.91)
2034	3,230,723 (2,626,148–3,835,298)	446.66 (364.18–529.13)	376,570 (364,615–388,525)	50.57 (47.42–53.72)
2035	3,221,876 (2,550,283–3,893,469)	443.95 (352.37–535.53)	376,568 (364,024–389,111)	50.24 (46.95–53.53)
Total population				
2022	7,887,971 (7,847,322–7,928,621)	553.24 (550.41–556.07)	834,978 (833,664–836,291)	58.65 (58.53–58.78)
2023	7,881,352 (7,763,814–7,998,890)	551.19 (543.08–559.29)	836,244 (833,136–839,351)	58.81 (58.46–59.15)
2024	7,865,975 (7,648,891–8,083,059)	548.09 (533.29–562.90)	836,720 (831,692–841,748)	58.98 (58.33–59.63)
2025	7,838,726 (7,512,365.–8,165,087)	544.06 (522.05–566.07)	836,704 (829,898–843,511)	59.15 (58.13–60.17)
2026	7,806,908 (7,366,411–8,247,405)	539.87 (510.43–569.31)	836,475 (828,141–844,810)	59.32 (57.87–60.76)
2027	7,777,111 (7,216,679–8,337,543)	536.01 (498.77–573.25)	836,220 (826,611–845,830)	59.48 (57.56–61.39)
2028	7,750,787 (7,062,230–8,439,344)	532.45 (486.83–578.06)	836,030 (825,352–846,707)	59.63 (57.215–62.05)
2029	7,726,041 (6,899,983–8,552,099)	528.94 (474.29–583.58)	835,925 (824,331–847,519)	59.77 (56.82–62.72)
2030	7,700,876 (6,728,303–8,673,449)	525.33 (461.06–589.60)	835,890 (823,481–848,298)	59.91 (56.41–63.41)
2031	7,674,709 (6,547,621–8,801,798)	521.62 (447.21–596.02)	835,895 (822,741–849,050)	60.03 (55.97–64.10)
2032	7,648,016 (6,359,239–8,936,793)	517.89 (432.90–602.89)	835,918 (822,062–849,774)	60.15 (55.51–64.79)
2033	7,621,392 (6,164,155–9,078,628)	514.20 (418.18–610.22)	835,941 (821,416–850,465)	60.26 (55.04–65.48)
2034	7,595,051 (5,962,735–9,227,367)	510.54 (403.06–618.02)	835,957 (820,790–851,125)	60.36 (54.55–66.16)
2035	7,568,882 (5,754,988–9,382,777)	506.88 (387.51–626.25)	835,966 (820,178–851,753)	60.45 (54.06–66.85)

### Discussion

Preterm birth, as an urgent global public health problem, is the main cause of neonatal incidence rate and mortality. In addition to the high risk of adverse health consequences, preterm birth also brings a huge economic burden (26–28). This study analyzed the long-term trends and risk factor attribution characteristics of preterm birth nursing rehabilitation needs and disease burden in Chinese newborns from 1990 to 2021 based on the GBD 2021 database. As a result, it was found that the demand for preterm birth rehabilitation needs and disease burden in Chinese newborns have decreased, with males bearing a higher burden than females. This may be largely attributed to the development of the social economy, advances in healthcare, and the government’s efforts in promoting women’s and children’s health. The attribution contribution of modifiable risk factors such as low birth weight and short pregnancy period is particularly prominent.

This study found that although preterm birth ASPR and ASYR in China have shown a slow downward trend (EAPC of −1.1% and −0.5%, respectively), this reflects significant progress in maternal and neonatal health in recent decades. The core of these improvements is initiatives such as Every Newborn Action Plan (ENAP) launched in 2014, which aims to eliminate preventable neonatal and maternal deaths by 2030, emphasizing the importance of improving postnatal care and reducing complications related to preterm birth. More research is needed on the content and quality of prenatal care to better understand how prenatal care can reduce the risk of preterm birth. Although the overall burden of preterm birth is decreasing, the burden of preterm birth in males has always been high, and the risk of preterm birth in males is higher than in females. The health department urgently needs to strengthen its attention to preterm birth in males. This is related to the fact that men are more likely to produce pro-inflammatory cytokines (29). This phenomenon is basically consistent with the global trend of liver cancer disease burden evolution (30). The inflammatory response of the placenta varies between genders. Male fetuses have a stronger pro-inflammatory response in their plasma, and placental cells also have a stronger response to inflammatory stimuli, producing more pro-inflammatory cytokines, while female fetuses tend to produce more anti-inflammatory factors. The differences in inflammatory response between fetuses of different genders may lead to variations in intrauterine infection rates and severity, which can increase the risk of premature birth and early-onset sepsis in males (31). In complex pregnancies such as preeclampsia, premature birth, and fetal growth restriction, the uterine artery pulsatility index is an important parameter for measuring the function of the uterine placental circulation. The gradient of the increase in the uterine artery pulsatility index in male fetuses is higher than that in females, indicating that the deterioration of uterine disc perfusion in male fetuses is more rapid in complex pregnancies. This difference may reflect differences in placental pathology between fetuses of different genders, as well as their impact on uterine placental blood flow resistance. Abnormal uterine artery blood flow during pregnancy may be one of the reasons why male fetuses have a relatively high risk of stillbirth and premature birth (32).

Implementing prenatal care policies is crucial for reducing the incidence of preterm birth (33). As shown in our Joinpoint model analysis, from 1990 to 2021, the ASPR (−0.74%) of preterm births in newborns showed a decreasing trend, with male ASPR (−0.82%) decreasing more than female ASPR (−0.65%); The ASYR (−0.22%) of newborns with preterm birth showed a decreasing trend, with males experiencing a higher decrease in ASYR (−0.24%) compared to females (−0.20%). This is consistent with our linkage analysis results, emphasizing the importance of structured prenatal care programs in improving maternal and child health outcomes. The decline in AAPC typically reflects advances in healthcare and the effectiveness of preventive interventions. Many strategies for preventing preterm birth, such as using progesterone to treat pregnant women with short cervix, actively managing pregnancy infections, and strengthening prenatal monitoring, mainly target identified high-risk pregnant women (34).

Research shows that short pregnancy, environmental particulate matter pollution, low birth weight, and household air pollution caused by solid fuels are still the main risk factors for preterm birth. Obtaining care that is risk appropriate is an effective way to reduce mortality associated with preterm birth. In rapidly urbanized areas, increased exposure to pollution and environmental degradation risks may lead to an increase in observed preterm birth rates, such as North Africa and the Middle East experiencing deteriorating air quality, which may result in an increase in preterm birth rates in these regions (35). Indoor air pollution caused by cooking with unclean fuels and indoor smoking has become an important factor in global mortality and incidence rate. For example, in Nigeria, mothers who use unclean cooking fuels face a higher risk of stillbirth (36). Low birth weight newborns have a higher risk of preterm birth. Low birth weight is an important indicator for evaluating malnutrition during pregnancy (37). Maternal malnutrition can be described by body size, and preterm birth may be caused by maternal emaciation due to reduced blood volume and uterine blood flow (38). Therefore, it is necessary to improve nutritional interventions for pregnant women to prevent low birth weight and preterm birth.

Epidemiological changes and population are the two main driving factors behind the changes in YLDs. Several studies have shown that the increase in immigration, often coming from areas with poor access to prenatal care, may put pressure on healthcare services and lead to an increase in preterm birth rates (39). In addition, the tightening policies and reduction in public healthcare funding in recent years may have limited access to basic maternal health services, thereby exacerbating these challenges. These economic and social pressures may have had a disproportionate impact on vulnerable groups, further leading to an observed increase in preterm birth rates (40). In addition, the increase in maternal age and related health complications may also play a role. Compared to young women, women over the age of 34 have a higher probability of preterm birth (41). In addition, our study used the ARIMA model to predict the long-term trend of preterm birth rehabilitation needs and disease burden in Chinese newborns from 2022 to 2035. The results showed that the preterm birth prevalence and ASYR showed a decreasing trend, while YLDs and ASYR show an upward trend. Therefore, specialized guidelines need to be developed to strengthen management and improve outcomes. Early identification of high-risk populations enables healthcare providers to implement interventions aimed at preventing or reducing the risk of preterm birth.

This study has several important limitations. First, the analysis relies entirely on modeled estimates from the GBD 2021 database. While GBD employs rigorous methodologies, its estimates are inherently subject to uncertainties arising from data sparsity, diagnostic variability, and modeling assumptions, particularly for conditions like preterm birth where accurate global and subnational data collection remains challenging. Second, the risk factor attribution was constrained to the four pre-defined factors quantified within the GBD framework. Other potentially significant factors, such as maternal nutrition, infection, stress, socioeconomic determinants, access to prenatal care, or specific clinical interventions, were not explicitly assessed in the decomposition or attribution analyses. Third, the projections to 2035 using ARIMA models assume that historical trends will continue linearly, which may not hold true given potential future changes in healthcare policies, environmental regulations, economic conditions, or unforeseen public health events. Finally, the national-level analysis masks potential significant subnational heterogeneity in preterm birth burden, risk factor exposure, and healthcare access across different regions within China. Future research incorporating primary, granular regional data and a broader range of modifiable risk factors is warranted.

## Conclusion

This study provides a comprehensive overview of the burden of premature birth in newborns in China from 1992 to 2021, the demand for neonatal preterm birth rehabilitation needs in China, as well as the number of cases with disease burden, ASPR and ASYR, have both decreased, while the number of YLDs has shown an upward trend, with a heavier burden on male preterm births. Due to the burden of preterm birth, postpartum care for mother and neonatal is needed with more care and intensity. Low birth weight and short-term pregnancy are the main risk factors for preterm birth. More attention should be paid to the management of risk factors, and targeted prevention and control strategies are urgently needed for specific causes of premature birth to reduce the disease burden. The future demand for neonatal preterm birth care and rehabilitation, as well as the prevalence of disease burden and ASPR, are showing a downward trend, but YLD and ASYR are showing an upward trend. Therefore, more comprehensive policies and preventive measures are still needed to effectively solve the problem of premature birth.

## Data Availability

The datasets presented in this study can be found in online repositories. The names of the repository/repositories and accession number(s) can be found at: https://ghdx.healthdata.org/gbd-2021.

## Footnotes

^1^ https://vizhub.healthdata.org/gbd-results/
